# Non-Hodgkin Lymphoma Developed Shortly after mRNA COVID-19 Vaccination: Report of a Case and Review of the Literature

**DOI:** 10.3390/medicina59010157

**Published:** 2023-01-12

**Authors:** Luigi Cavanna, Sergio Ottavio Grassi, Livia Ruffini, Emanuele Michieletti, Egidio Carella, Dante Palli, Adriano Zangrandi, Nicola Inzerilli, Patrizia Bernuzzi, Camilla Di Nunzio, Chiara Citterio

**Affiliations:** 1 Department of Oncology and Hematology, ASL Piacenza, Via Taverna 49, 29121 Piacenza, Italy; 2 ASST Lodi, 26900 Lodi, Italy; 3 Nuclear Medicine Division, Azienda Ospedaliero-Universitaria di Parma, 43126 Parma, Italy; 4 Department of Radiology, ASL Piacenza, Via Taverna 49, 29121 Piacenza, Italy; 5 Surgey and Breast Unit, Guglielmo da Saliceto Hospital, Via Taverna 49, 29121 Piacenza, Italy; 6 Pathology Unit, ASL Piacenza, Via Taverna 49, 29121 Piacenza, Italy

**Keywords:** COVID-19, vaccination, lymphoma

## Abstract

We report on a 66-year-old man who presented with a right axillary lymphadenopathy approximately 10 days after receiving the third dose of the BNT162b2 vaccine. The lymphadenopathy gradually enlarged, and physical examination and ultrasound (US) revealed one right axillary 6.99 cm and one right supraclavicular 2.36 cm lymphadenopathy. Histologic examination of the right axillary nodule revealed anaplastic large-cell lymphoma that was ALK negative and CD30 positive. A total body computerized tomography (CT) scan, positron emission tomography (PET) and bone-marrow biopsy showed a stage-II non-Hodgkin lymphoma (NHL). The patient was treated with chemotherapy and a scheme of Brentuximab Vedotin, Cyclophosphamide, Doxorubicin and Prednisone (BV-CHP) for six cycles and is now well and in complete remission. The revision of the literature revealed eight additional cases of NHL developed shortly after COVID-vaccination. There were four cases of diffuse large-B-cell lymphoma (DLBCL) (one in a patient who was a heart transplant recipient and developed an Epstein–Bar-virus-positive DLBCL), one case of extranodal NK/T-cell lymphoma, one patient with subcutaneous panniculitis-like T-cell lymphoma, one case of marginal zone B-cell lymphoma and one primary cutaneous anaplastic large-cell lymphoma (PC-ALCL). In five cases, the lymphoma developed after BNT162b2 mRNA vaccination, including one case after ChAdOx1 nCOV-19, one case after the adenovirus type 26 (Ad26) vaccine and one after mRNA-1273/Spikevax (ModernaTX). We are aware that the link between COVID-19 vaccination and lymphoma most likely is a chance phenomenon, and that COVID-19 vaccines represent very efficient products for many people around the world. However, we believe that clinical events, even if only temporally associated with novel treatments or novel vaccines, should be reported for the benefit of the patients and the scientific community.

## 1. Introduction

Axillary and cervical lymphadenopathy related to recent vaccination is a well-known event following vaccines such as those for smallpox, influenza, human papilloma virus and other vaccines [1,2]. More recently, COVID-19 vaccination was identified as a cause of inflammatory unilateral axillary and cervical adenopathy [3,4]. The median duration of adenopathy reported after mRna-1273 (Moderna) and BNT162b2 (Pfizer) vaccine administration ranges from 1–2 days to approximately 10 days in duration; however, in some cases, the lymphadenopathies persisted for more than 1 month after vaccination [3,4]. The differential diagnosis of lymphadenopathies reactive with other types of lymphadenopathies, such as lymphoma, is very important. Generally, post-vaccine-reactive lymphadenopathy shows a progressive decrease within a number of days after vaccination [5].

Herein, we discuss a case of right axillary lymphadenopathy developed 10 days following the third dose of BNT162b2 vaccination, which was suspected to be a reactive lymphadenopathy. However, the mass gradually enlarged, and a biopsy of the nodule demonstrated non-Hodgkin lymphoma (NHL). In addition, we performed a review of the literature, and here, we report the results of this review. Written informed consent for the publication of details and images of the case report was obtained from the patient and is available for verification by the handling editor if needed.

## 2. Case Report

The patient reported on here is a 66-year-old white man with no previous history of disease. In January 2021, he received the first dose of the BNT162b2 (Pfizer) vaccine, and after one month, he received a second dose of the BNT162b2 vaccine. In October 2021, he received a booster dose of the same vaccine in the right arm, and subsequently, he showed a mobile and painful right axillary lymphadenopathy, which arose approximately 10 days after the administration of the vaccine booster, with no other clinical history of interest.

**Figure 1:**
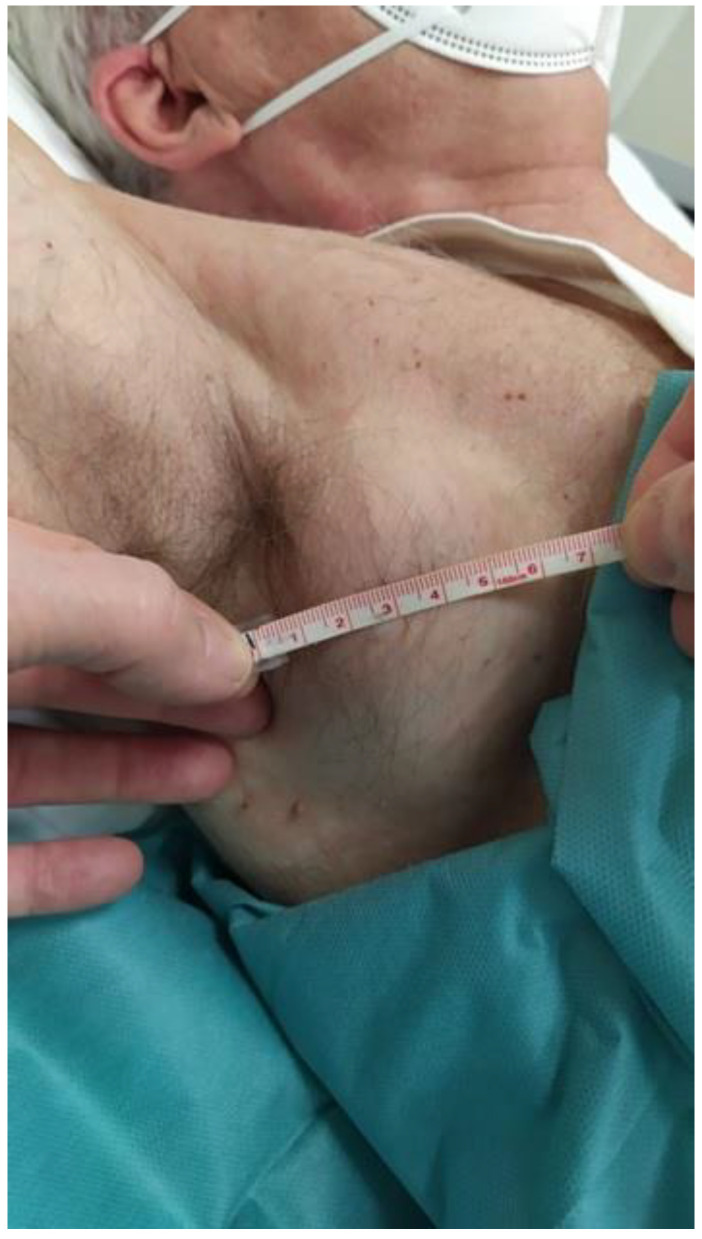
Physical examination revealed a large right axillary adenopathy.

The patient presented to our outpatient clinic 3 months later with enlarged right axillary adenopathy that was confirmed upon physical examination, which also revealed an ipsilateral supraclavicular lymphadenopathy (Figure 1).

**Figure 2:**
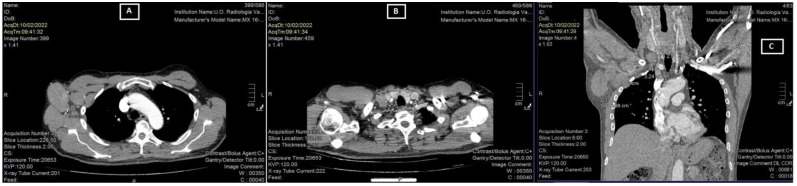
CT scan revealed two lymphadenopathies: right axillary 6.99 cm (**A**) and supraclavicular 2.36 cm (**B**), and multiple adenopathies in the coronal section (**C**).

The lymphadenopathies were painful and mobile. A total body computed tomography (CT) examination confirmed the two lymphadenopathies, measuring 6.99 cm and 2.36 cm, respectively (Figure 2). 

**Figure 3:**
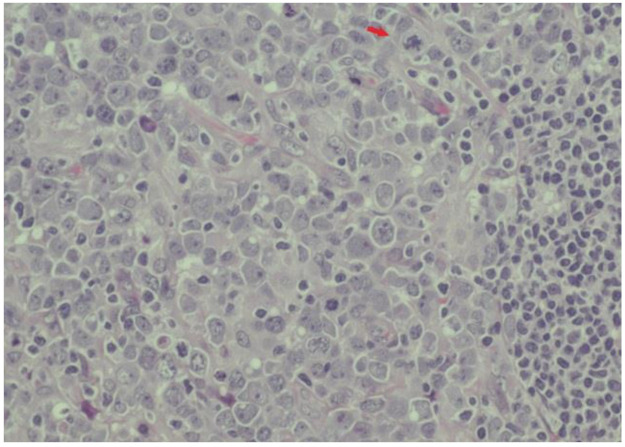
Histologic sample from axillary lymphadenectomy: tumor cells are very large (compared with the normal lymphocytes on the right) with highly atypical nuclei and macronucleoli. Apoptotic bodies and mitoses are frequent, including atypical mitoses (red arrow).

**Figure 4:**
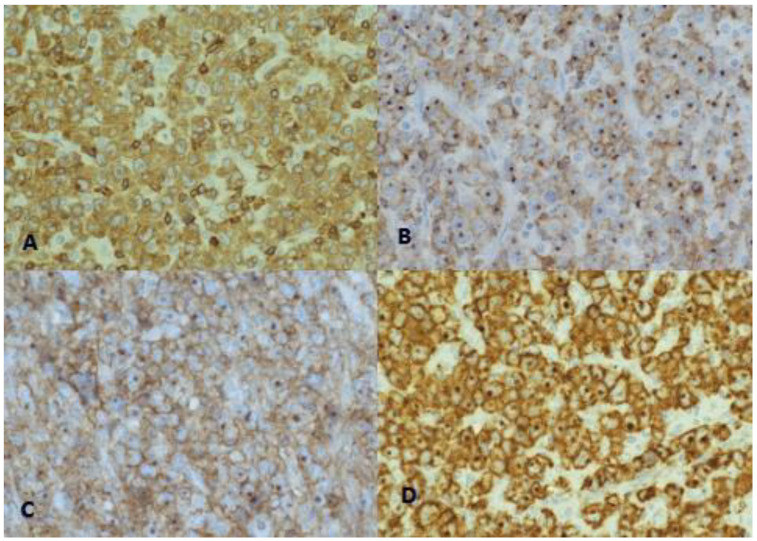
Lymphoma cells are positive for T-cell markers: CD3 (**A**), CD2 (**B**) and CD4 (**C**). They are also diffusely and strongly positive for CD30, with a membranous and dot-like pattern (**D**). ALK protein was negative (not shown).

A US-guided, fine-needle aspiration biopsy of the axillary lymphadenopathy was carried out, and suspicion of NHL was suggested. A surgical biopsy of the right axillary adenopathy revealed anaplastic large-cell lymphoma that was ALK negative and CD30 positive, with the presence of elevated Ki-67 proliferation antigen (90%) (Figure 3 and Figure 4).

**Figure 5:**
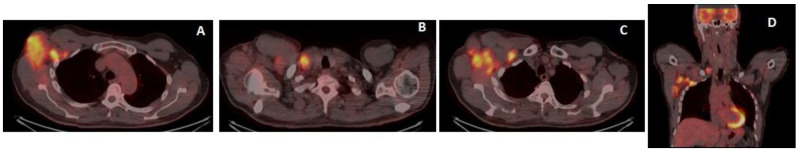
18-FDG-PET/CT at baseline (axial and coronal sections), increased tracer uptake in the right axillary adenopathy mass (SUV max 9.7) (**A**), in the right supraclavicular adenopathy (SUV max 7.6) (**B**) and in multiple axillary adenopathies (level I–III) (**C**,**D**).

18F-Fluorodeoxyglucose positron emission tomography/CT (18-FDG-PET/CT) showed increased tracer uptake in the right axillary adenopathy, in the right supraclavicular adenopathy and in multiple axillary adenopathies (Figure 5).

**Figure 6:**
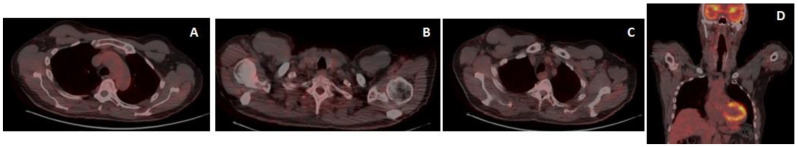
Restaging 18-FDG-PET/CT after chemotherapy showed the complete metabolic response of all the involved sites (**A**–**D**).

A bone marrow biopsy was performed, a diagnosis of stage-II NHL was made, and the patient underwent monoclonal antibody anti-CD30 Brentuximab Vedotin in association with CHP chemotherapy (Cyclophosfamide, Adriblastine, Prednisone) for six cycles from March to July 2022, with complete remission after three cycles (early PET). No particular signs of toxicity were observed during treatment, which was well-tolerated. The patient is on follow-up, with a good health condition and persistent complete remission, as confirmed by PET/CT restaging at the end of the treatment (Figure 6).

## 3. Revision of the Literature

### Methods

Case reports of lymphoproliferative disorders related to the COVID-19 vaccine were systematically researched using databases including PUBMED, SCOPUS, Science Direct and EBSCO Host.

The general research terms used included the following: “COVID-19, SARS-CoV-2, COVID-19 vaccine, mRNA vaccine, malignancy, neoplasm, cancer, lymphoma, lymphoproliferative disease”.

The research was carried out on 15 November 2022 and was limited to literature in English. The research was conducted independently by two investigators (LC and CC).

## 4. Results of the Literature Revision

Eight patients who developed NHL after COVID-19 vaccination were identified, including four males and four women. Five patients were vaccinated with the BNT162b2 vaccine (Pfizer), one with the ChAdOx1 nCOV-19 vaccine (AstraZeneca, Cambridge, UK), one with mRNA-1273/Spikevax (ModernaTX) and one patient with the recombinant replication-incompetent adenovirus type 26 (Ad26) viral-vector-based COVID-19 vaccine (Janssen Pharmaceuticals, Beerse, Belgium) (Table 1) [6,7,8,9,10,11]. Five patients had B-cell lymphoma and three had T-cell lymphoma, while four patients were diagnosed with diffuse large-B-cell lymphoma (DLBCL). One patient had extranodal NK/T-cell lymphoma [7], and one patient, a heart transplant recipient, developed a rapidly growing Epstein–Barr-virus (EBV)-positive diffuse large-B-cell lymphoma 7 days after receiving the first dose of the ChAdOx1 nCOV-19 vaccine [8]. One patient had a subcutaneous panniculitis-like T-cell lymphoma (SPTCL) [9], one had an extranodal marginal zone lymphoma (EMZL) [10] and one primary cutaneous anaplastic large-cell lymphoma (PC-ALCL) [11]. The lymphoproliferative disorder developed shortly after the vaccination, within 1 to 10 days following vaccination (Table 1). Four of these eight patients were treated with systemic treatment, one was treated with radical surgery and radiation therapy, one was treated with systemic treatment and radiation therapy, one was treated through the watchful waiting program for comorbidities and the last one showed spontaneous, almost total remission without treatment after 6 weeks of follow-up.

## 5. Discussion

Reactive post-vaccination lymphadenopathy has been described in relation to multiple vaccine types [1,2] in most cases. Lymphadenopathies secondary to vaccination are ipsilateral axillary or cervical, corresponding to the site of vaccination. First unilateral axillary lymphadenopathy post-COVID-19 vaccination was reported based on imaging scans in January 2021 among women subjected to ultrasound examination of the breast [12]. Subsequently, several cases of benign unilateral axillary lymphadenopathy after COVID-19 vaccination were reported [13,14,15], with a transient character [12,13,14,15].

It must be emphasized that unilateral axillary lymphadenopathy is common after both mRNA-1273 (Moderna) and BNT 162b2 (Pfizer) [3,4].

Mizutani M. and colleagues [6] reported two cases of diffuse large-B-cell lymphoma that developed shortly after BNT162b2 COVID-19 vaccination. The axillary lymphadenopathies developed 1 day after the first dose of BNT162b2 vaccination in a 67-year-old Japanese man and 1 day after the second dose of BNT162b2 vaccination in an 80-year-old Japanese woman. The patients were treated with chemotherapy combined with rituximab, showing response to the treatment. More recently, two additional cases of malignant lymphoma were reported [7]. In one case, a 58-year-old female patient presented with a left cervical mass of 4 cm in diameter one week after the second dose of BNT162b2. A histopathological examination revealed DLBCL, and the patient was treated with radical surgery and radiation therapy, with response to the treatment. In the second case, a 53-year-old male developed a painless ulcer on the mucosal surface of the upper lip three days after the first dose of the BNT162b2 COVID-19 vaccine. Subsequently, the ulcer worsened, and a biopsy revealed extranodal NK/T-cell lymphoma that was treated with chemotherapy and radiotherapy, with response to the treatment. A 51-year-old man who previously received heart transplantation developed a mediastinal mass approximately 52 × 50 × 42 mm one week after the first dose of the ChAdOx1 nCOV-19 vaccine (Astrazeneca), administrated to the right deltoid muscle. The pathologic examination revealed EBV-positive DLBCL. The patient was in treatment with an immunosuppressant regimen for transplantation, and the final diagnosis was consistent with post-transplant lymphoproliferative disorder (PTLD). He underwent debulking surgery combined with rituximab and a 50% reduction in the immunosuppressant dosage and showed response to the treatment [8]. A case of a very rare SPTCL lymphoma was recently described in a previously healthy 28-year-old woman, and it developed a few days after receiving the primary dose of the Ad26 viral-vector-based COVID-19 vaccine (Janssen Pharmaceuticals). The patient was treated with cyclosporine combined with prednisone, with complete remission [9]. An 80-year-old Japanese woman developed a right temporal mass that suddenly appeared on the morning after she received her first COVID-19 vaccine (BNT162b2) in her left deltoid muscle. Since the temporal mass increased to 68.3 × 17.1 mm 16 weeks after the first vaccination, a biopsy was performed, and a diagnosis of EMZL was made. Chemotherapy was suggested to the patient; however, she preferred careful monitoring [10]. A 76-year-old man presented with a large erythematous rounded tumor of 6 cm in diameter on his right arm ten days after the application of the booster dose of the mRNA-1273 SARS-CoV2 vaccine, with a diagnosis of PC-ALCL. After 6 weeks of follow-up, the lesion showed spontaneous, almost total remission without treatment [11].

The majority of the cases described above and the case of our patient reported here showed similar features, including the short time between vaccination and the development of lymphoma, ranging from 1 to 10 days; the pathologic feature, being B-cell lymphoma in 5/8 cases; and the large dimensions of the lymphadenopathy, ranging from 4 to 6 cm. To the best of our knowledge, this is the first review of malignant lymphoma diagnosed shortly after COVID-19 vaccination. Previous reports showed a progression or pseudo-progression of the disease in patients with a pre-existing lymphoma [16,17,18,19,20]. On the other hand, it must be emphasized that spontaneous tumor regression after COVID-19 vaccination was reported in a patient with metastatic myoepithelial carcinoma of the left parotid [21] and in a patient with cutaneous anaplastic large-cell lymphoma [22]. It is most likely that lymphoma is a chance phenomenon in these cases and that there is an absence of any evidence to support the notion that COVID-19 vaccination is linked in any way to an increased risk of lymphoma incidence. This study is a case report and is not designed to address the question of whether COVID-19 vaccination is linked to the risk of lymphoma. However, as reported by Zamfir MA. et al. [7] and Goldman S. et al. [16], COVID-19 vaccines showed a potent stimulation of the immune cells, especially the T follicular helper (TFH) cells, resulting in persistent germinal center B-cell responses. For these reasons, our case report and the literature review suggest that the appearance of post-vaccine lymphadenopathy should be followed as recommended [4,12,13], and in cases of suspicion of lymphoproliferative disorder, a histopathological examination should be carried out to obtain a definite diagnosis. In fact, the clever physician should not take any lymphadenopathy lightly and perhaps keep in mind the possibility of a more sinister process, such as lymphoma. Patients should thus always be followed-up to ensure that any suspected reactive lymphadenopathy resolves over time. Otherwise, it is necessary to consider further work-up, including an excision biopsy.

In conclusion, according to the scientific knowledge, we are aware that the COVID-19 vaccines represent very efficient products with a favorable benefit–risk ratio. However, we believe that it is mandatory to communicate all events to the scientific community, even those only temporally linked with novel treatments or vaccines such as the COVID-19 vaccines.

## Figures and Tables

**Table 1:** Review of the literature: malignant lymphoma diagnosed in the context of the mRNA COVID-19 vaccination (Ad26: adenovirus type 26, ALCL: anaplastic large-cell lymphoma, DLBCL: diffuse large-B-cell lymphoma, EBV: Epstein–Barr virus, EMZL: extranodal marginal zone lymphoma, PC-ALCL: primary cutaneous anaplastic large-cell lymphoma, SPTCL: subcutaneous panniculitis-like T-cell lymphoma). * The two previous vaccination doses were BNT162b2.

Case N.	Gender/Age (Year) (Reference)	Time from Vaccination to Onset of Lymphoproliferative Disorder	Histopathological Examination	Type of COVID-19 Vaccine	Site and Diameter of Lymphadenopathy	Treatment of Lymphoma
1	M/67 [6]	1 day after 1 dose	DLBCL	BNT162b2	Left axilla 6.0 cm	Chemotherapy plus rituximab
2	F/80 [6]	2 days after 1 dose	DLBCL	BNT162b2	Left axilla 4.1 cm	Chemotherapy plus rituximab
3	F/58 [7]	7 days after 2 dose	DLBCL	BNT162b2	Left cervical area 4 cm	Radical surgery plus radiotherapy
4	M/53 [7]	3 days after 1 dose	Extranodal NK/T-cell lymphoma	BNT162b2	Erosive lesions upper lip up to 5 mm	Chemotherapy plus radiotherapy
5	M/51 [8]	7 days after 1 dose	EBV-positive DLBCL	ChAdox1 nCOV-19	Mediastinal mass 5 cm	Rituximab
6	F/28 [9]	“A few days after 1 dose”	SPTCL	Ad26 viral-vector-based	Injection site, upper arm	Cyclosporine plus prednisone
7	F/80 [10]	1 day after 1 dose	EMZL	BNT162b2	Right temporal mass	No treatment
8	M/76 [11]	10 days after the booster dose	PC-ALCL	mRNA-1273 *	Right arm upper-external surface 6 cm	No treatment

## Data Availability

The data presented in this study are available on request from the corresponding author.
